# Microbiological and Clinical Characteristics of Hypermucoviscous *Klebsiella pneumoniae* Isolates Associated with Invasive Infections in China

**DOI:** 10.3389/fcimb.2017.00024

**Published:** 2017-02-01

**Authors:** Yinjuan Guo, Shanshan Wang, Lingling Zhan, Ye Jin, Jingjing Duan, Zhihao Hao, Jingnan Lv, Xiuqin Qi, Liang Chen, Barry N. Kreiswirth, Liangxing Wang, Fangyou Yu

**Affiliations:** ^1^Department of Laboratory Medicine, the First Affiliated Hospital of Wenzhou Medical UniversityWenzhou, China; ^2^Public Health Research Institute Tuberculosis Center, New Jersey Medical School, Rutgers UniversityNewark, NJ, USA; ^3^Department of Respiratory Medicine, the First Affiliated Hospital of Wenzhou Medical UniversityWenzhou, China

**Keywords:** *Klesiella pneumonia*, hypermucoviscosity, characteristics, epidemiology

## Abstract

A distinctive syndrome caused by hypermucoviscous *Klebsiella pneumoniae* (HMKP) including pyogenic liver abscess (PLA) is now becoming a globally emerging disease. In the present study, 22.8% (84/369) of *K. pneumoniae* clinical isolates associated with various types of invasive infections were identified as HMKP, with 45.2% associated with PLA. Multivariate regression analysis showed that male patients with 41–50 years, PLA, diabetes mellitus, and hypertension were independent risk factors for HMKP infections. K2 (42.9%, 36/84) was the most common capsular serotype among HMKP isolates, followed by K1 (23.8%, 20/84). Seventy-five percentage of K1 HMKP isolates were associated with PLA, while K2 HMKP isolates accounted for more types of invasive infections. The positive rates of *iutA, mrkD, aerobactin, iroN*, and *rmpA* among HMKP isolates were significantly higher than those among non-HMKP isolates (*p* < 0.05). There was a correlation between *magA, ybtS, alls*, and *wcaG* and K1 isolates. Interestingly, *mrkD* was exclusively detected among HMKP (32.1%, 27/84) and K2 isolates (65.9%, 27/41). All K1 and K2 HMKP and non-HMKP isolates were positive for *rmpA*. *Aerobactin* was found among 95.0 and 97.5% of K1 and K2 isolates. ST23 was found to be the most prevalent ST among 69 HMKP isolates with K1, K2, K5, K20, and K57 (27.5%, 19/69) and was only found among K1 isolates. ST65 was the second most prevalent ST (26.1%, 18/69) and was also only found among K2 isolates. ST23-K1 HMKP isolates (84.2%, 16/19) were associated with PLA, while ST65-K2 isolates were correlated with more types of infections relative to ST23-K1 isolates. PFGE results showed that the homology of 84 HMKP isolates was diverse. Only five PFGE clusters with more than 75% similarity accounted for more than three isolates. These five PFGE clusters only accounted for 35 (41.7%, 35/84) isolates. In conclusion, our study first found that hypertension and male patients with 41–50 years old were independent risk factors. The composition of ST types and PFGE clusters among *K. pneumoniae* K2 isolates was more diverse than K1 isolates. K1 and K2 HMKP isolates had respective specific profiles of virulence-associated genes.

## Introduction

*Klebsiella pneumoniae* is a frequently-isolated and well-established bacterial pathogen responsible for numerous infections in hospitals and long-term care facilities worldwide (Shon et al., 2013). Infections caused by *K. pneumoniae* can involve lung, urinary tract, surgical sites, abdominal cavity, intra-vascular devices, soft tissues, and subsequent bacteremia (Shon et al., 2013). Between the mid-1980s and 1990s, reports from Taiwan described a distinctive syndrome of community-acquired invasive *K. pneumoniae* infections, mainly in the form of pyogenic liver abscesses (PLA; Liu et al., 1986; Cheng et al., 1991; Wang et al., 1998). These infections are often complicated by devastating metastatic infections including endophthalmitis and meningitis in younger and healthy individuals (Struve et al., 2015). The *K. pneumoniae* strains associated with these serious infections are defined as hyper-virulent (hypermucoviscous) *K. pneumonia* (hvKP), with a distinct hypermucoviscosity phenotype when grown on agar plates (Struve et al., 2015). Although these infections were reported initially within Southeast Asia including Taiwan, South Korea, and Japan, an increasing number of cases were reported from North America, Europe, South America, the Middle East, Australia, Africa and China, indicates that this unique syndrome is becoming a globally emerging disease (Fang et al., 2005; Siu et al., 2012; Shon et al., 2013; Bialek-Davenet et al., 2014; Yang et al., 2014; Struve et al., 2015; Prokesch et al., 2016). A combination of clinical and bacterial phenotypic features is used for distinguishing hvKP from classic *K. pneumoniae* (cKP) strains. The hvKP isolates characteristically express a distinct hypermucoviscosity phenotype determined by string test when grown on agar plates and most of these isolates belong to capsular serotype K1 and K2 (Fang et al., 2007; Shon and Russo, 2012; Cheng et al., 2015). A number of putative virulence factors, mainly including mucoviscosity-associated gene A (*magA*) and regulator of mucoid phenotype A (*rmpA*), have been found to be associated with hvKP (Fang et al., 2004; Yu et al., 2006). Recently, aerobactin accounting for increased siderophore production was found to be a major virulence determinant and new defining trait for hvKP (Russo et al., 2014). A multiplex PCR assay targeting seven virulence factors and the *wzi* gene specific for the K1 and K2 *K. pneumoniae* was developed for the surveillance of emerging highly virulent strains (Compain et al., 2014; Russo et al., 2014). Alarmingly, increasing studies reported that multi-drug-resistant, even carbapenem-resistant hvKP isolates have been emerged (Yang et al., 2014; Yao et al., 2015; Zhang et al., 2015), which is becoming an important threat to public health. Recently, many studies from China described the prevalence, clinical presentations and epidemiology of hvKP isolates (Liu et al., 2014; Yang et al., 2014; Qu et al., 2015; Yao et al., 2015; Sun et al., 2016; Zhao et al., 2016). However, the limitations of these studies included limited number of *K. pnuemoniae* involved, short span of investigation and focusing a specific infection. In the present study, we investigated the prevalence, clinical characteristics, and molecular epidemiology of hypermucoviscous *Klebsiella pneumoniae* (HMKP) isolates with hypermucoviscosity phenotype determined by the string test among 369 *K. pneumoniae* isolates associated with various invasive infections from January 2013 to October 2015.

## Materials and methods

### Collection and identification of *K. pneumoniae* clinical isolates

From January 2013 to October 2015, a total of 369 *K. pneumoniae* isolates were consecutively isolated from the specimens of patients with various invasive infections at the first Affiliated Hospital of Wenzhou Medical University located in Wenzhou, east China. The *K. pneumoniae* isolates from the specimens of respiratory tract, urinary tract, and intestinal tract were excluded in this investigation, because it is difficult to discriminate invasive isolates from colonizing isolates. *K. pneumoniae* isolates were identified by Gram-staining and a VITEK-2 automated platform (bioMérieux, Marcy l'Etoile, France). *Staphylococcus aureus* ATCC25923, *Escherichia coli* ATCC25922, and *Pseudomonas aeruginosa* ATCC87253 were used as control strains for the identification of bacterial clinical isolates. Acquiring the information in the medical records of the patients and the *K. pneumoniae* isolates for research purposes were approved by the ethical committee of the first Affiliated Hospital of Wenzhou Medical University. Informed consents were obtained from the patients involved.

### Antimicrobial susceptibility testing

The susceptibility of the *K. pneumoniae* clinical isolates included to clinically often used antimicrobial agents was also determined using VITEK-2 automated platform (bioMérieux, Marcy l'Etoile, France) in accordance with the manufactory' s instructions. *Escherichia coli* ATCC25922 was used as control strain for determining the antimicrobial susceptibility for *K. pneumoniae* clinical isolates.

### Phenotypical identification of HMKP isolates

The *K. pneumoniae* invasive isolates with hypermucoviscosity phenotype determined by string test described previously were identified as HMKP (Shon et al., 2013). Briefly, when an inoculation loop or needle was able to generate a viscous string >5 mm in length by stretching *K. pneumoniae* colonies on Columbia blood agar plate (BIO-KONT, Wenzhou, China), the isolate was considered to be positive for the string test and was defined as HMKP.

### Capsular serotyping and detection of virulence-associated genes

K1, K2, K5, K20, K54, and K57 capsular serotypes were identified by PCR as described previously (Fang et al., 2007; Turton et al., 2010). Seventeen virulence-associated genes, including *ybtS, ureA, uge, wabG, ycf*, *entB, iutA, vatD, magA, fimH, mrkD, aerobactin, iroN, kfuB, rmpA, wcaG*, and *alls*, were determined by PCR using primers described previously (Yu et al., 2006, 2008; Turton et al., 2010; Candan and Aksöz, 2015) for all HMKP and 95 selected randomly non-HMKP isolates. The isolates with virulence-associated genes determined by PCR and DNA sequencing were selected as positive control strains for the subsequent PCR experiments.

### Multi-locus sequence typing (MLST)

MLST was performed on HMKP isolates by amplifying internal fragments of the seven standard housekeeping loci (*gapA, infB, mdh, pgi, phoE, rpoB*, and *tonB*) as described previously (Diancourt et al., 2005). Sequence types (STs) were identified using the online database on the Pasteur Institute MLST website (http://www.pasteur.fr/recherche/genopole/PF8/mlst/Kpneumoniae.html).

### Pulsed-field gel electrophoresis (PFGE)

PFGE was performed on 84 HMKP isolates using XbaI digestion for 12 h at 37°C and electrophoresis for 20 h at 14°C, 120 angle, with switch times of 6 and 36 s at 6 V/cm, Bio-Rad CHEF III system. Comparison of the PFGE patterns was performed with Bionumerics software (Applied Maths, Sint-Martens-Latem, Belgium) using the Dice Similarity coefficient. More than 75 percentage of similarity was used for the threshold for clustering in PFGE pattern analysis.

### Statistical analysis

Statistical analysis was performed using SPSS statistical software (version 19, IBM SPSS Statistics). The χ^2^ or Fisher's exact tests were used for categorical variables. Logistic regression with univariate model and multivariate model was used to identify variables associated with HMKP infections. *P* < 0.05 was considered statistically significant. Diabetes, hypertension, liver abscess, sex, age, and leukemia were used for the logistic regression model.

## Results

### Prevalence and clinical characteristics of HMKP isolates

Three hundred and sixty-nine *K. pneumoniae* invasive isolates were obtained from clinical specimens including 129 blood (35.0%), 133 pus (35.4%), 51 drainage (13.6%), 2 pleural effusion (0.5%), 20 bile (5.3%), 8 ascites (2.1%), 9 tissue (2.4%), 13 catheter tip (3.5%), and 3 cerebrospinal fluid (0.8%). ≤60 and >60-year old patients accounted for 162 (43.9%) and 207 patients (56.1%). The male and female patients accounted for 62.1% (229) and 37.9% (140). Among 369 *K. pneumoniae* invasive isolates, 84 (22.8%) were positive for the string test and were identified as HMKP. The proportions of HMKP isolates among *K. pneumoniae* invasive isolates in 2013, 2014, and 2015 were 23.2% (23/99), 24.3% (33/136), and 20.9% (28/134), respectively, with a stable HMKP prevalence.

The proportions of HMKP among *K. pneumoniae* isolates from different specimens were as follows: blood, 17.1% (22/129); pus, 32.3% (43/133); drainage, 23.5% (12/51); pleural effusion, 100% (2/2); bile, 5% (1/20); ascites, 25% (2/8); tissue, 11.1% (1/9); and catheter tip, 7.7% (1/13), respectively. These HMKP isolates were associated with various types of invasive infections including PLA (37, 44.0%), bacteremia (21 including seven associated with PLA, 25.0%), abdominal infections (13, 14.8%), skin and soft tissue infections (SSTIs; nine including two causing necrotic fascilitis, 10.7%), and lung abscess (4, 4.8%).

### Risk factors for invasive HMKP infections

The clinical characteristics of patients with HMKP and non-HMKP infections were showed in Table 1. In the present study, there were no differences between the proportions of HMKP among male and female patients regardless of age, as well as among patients with >60 years old and ≤60 years old regardless of sex. Further investigation found that HMKP prevalence in male patients with 41–50 years old was 40.6% (13/32), while no HMKP was found in female patients with the same age. Univariate analysis revealed that male patients with 41–50 years (OR = 2.554) was found to be statistically significant risk factors for HMKP infections. Multivariate regression analysis further demonstrated that male patients with 41–50 years [aOR = 2.482, 95%CI(1.069–5.760)] was an independent risk factor for HMKP infections (Table 2). Diabetes mellitus was found among 29.8% (25/84) of patients with HMKP infections and 16.8% (48/285) of patients with HMKP infections. Multivariate analysis showed diabetes mellitus [aOR = 3.417, 95%CI(1.632–7.155)] was an independent risk factor for HMKP infections (Table 2).

**Table 1:** **Clinical characteristics of HMKP and non-HMKP isolates**.

	**HMKP (*n* = 84)**	**non-HMKP (*n* = 285)**	***P-*****values**
Male	54 (64.3%, 54/84)	175 (70.3%, 175/285)	0.632
≤60 years old	39 (46.4%, 39/84)	123 (43.2%, 123/285)	0.592
**Male**	**32 (82.1%, 32/39)**	**79 (64.2%, 79/123)**	**0.037**
>60 years old	45 (53.6%, 45/84)	162 (58.9%, 162/285)	0.592
Male	22 (48.9%, 22/45)	96 (59.3%, 96/163)	0.214
≤40 years old	8 (14.8%, 8/54)	34 (11.9%, 34/285)	
Male	5 (62.5%, 5/8)	18 (52.9%, 18/34)	0.709
**41–50 years old**	**13 (15.5%, 13/84)**	**32 (11.2%, 32/285)**	
**Male**	**13 (100%, 13/13)**	**19 (59.4%, 19/32)**	**0.009**
51–60 years old	18 (21.4, 18/84)	52 (18.2%, 52/285)	
Male	14 (77.8%, 14/18)	40 (76.9%, 40/52)	1.000
61–70 years old	27 (32.1%, 27/84)	72 (25.3%, 72/285)	
Male	13 (48.1%, 13/27)	43 (59.7%, 43/72)	0.301
71–80 years old	14 (16.7%, 14/84)	63 (22.1%, 63/285)	
Male	7 (50%, 7/14)	35 (55.6%, 35/62)	0.706
>80 years old	4 (4.8%, 4/84)	27 (9.5%, 27/285)	
Male	2 (50%, 2/4)	17 (63.0%, 17/27)	0.63
**Diabetes**	**25 (29.8)%, 25/84**	**48 (16.8%, 48/285)**	**0.009**
**Hypertension**	**10 (13.1%, 10/84)**	**2 (0.7%)**	<**0.001**
**STC**^a^	**2 (2.4%, 2/84)**	**58 (9.2%, 26/285)**	**0.086**
**Liver abscess**	**38 (45.2%, 38/84)**	**43 (15.1%, 43/285)**	<**0.001**
**Leukemia**	**1 (1.2%, 1/84)**	**27 (9.5%, 27/285)**	<**0.001**
Liver disease^b^	5 (5.96%, 5/84)	38 (13.4%, 38/285)	0.064

a *STC, solid tissue cancer*.

b *Not including liver abscess*.

*Bold values indicate parameters with P* < 0.05 *which was considered statistically significant*.

**Table 2:** **Regression analysis of variables associated with HMKP infections**.

	**OR (95% CI)**	***p***	**aOR (95% CI)**	***p***
Diabetes	6.262 (3.210–12.216)	0.000	3.417 (1.632–7.155)	0.001
Hypertension	12.658 (3.397–47.16)	0.000	7.333 (1.748–30.766)	0.006
Liver abscess	4.760 (2.773–8.170)	0.000	3.648 (2.045–6.507)	0.000
41–50-years old males	2.554 (1.203–5.420)	0.012	2.482 (1.069–5.760)	0.034
Leukemia	0.102 (0.014–0.760)	0.007	0.190 (0.025–1.449)	0.109

### Capsular serotyping of HMKP and non-HMKP isolates

Of 84 HMKP isolates, capsular serotype K1 (23.8%, 20/84), K2 (42.9%, 36/84), K5 (2.4%, 2/84), K20 (4.8%, 4/84), and K57 (9.6%, 8/84) were identified, while K54 was not found in any of the isolates tested. Fourteen HMKP isolates (16.7%) were not typed successfully and were defined as K-non-typable isolates. K1 prevalence among HMKP isolates (23.8%, 20/84) was similar to that among non-HMKP isolates (23.2%, 22/95). However, more HMKP isolates belonged to K2 relative to non-HMKP isolates (6.3%, 6/95). Among 38 HMKP isolates associated with PLA, 15 (37.5%), 14 (35.0%), 4 (10.0%), and 1 (2.5%) belonged to K1, K2, K57, and K20, respectively. The capsular serotypes of 22 HMKP isolates associated with bacteremia were K1 (18.2%, 4/22), K2 (22.7%, 5/22), K57 (18.2%, 4/22), K20 (9.1%, 2/22), and K2 (4.5%, 1/22) and K-non-typable capsular serotype (27.3%, 6/22). 6, 2, and 1 of 9 HMKP causing SSTIs belonged to K2, K1, and K5. Among 13 isolates associated with abdominal infections, 3, 2, 2, and 1 belonged to K2, K20, K57, and K1, respectively. Three K2 and one K57 were associated with lung abscess.

### Antimicrobial resistance among HMKP and non-HMKP isolates

HMKP isolates were more susceptible to clinically often used antimicrobial agents relative to non-HMKP isolates (Table 3). All HMKP and non-HMKP isolates were resistant to ampicillin. The resistance rates of 84 HMKP isolates to cefotetan, cefepime, ertapenem, imipenem, piperacillin/tazobactam, and trimethoprim-sulfamethoxazole were 4.8% (4/84). 7.1% (6/84) HMKP isolates were resistant to aztreonam, ceftazidime, and ciprofloxacin. 5.9% (5/84) HMKP isolates were resistant to ciprofloxacin, levofloxacin, and gentamicin. 8 (9.5%), 7(8.3%), 3(3.6%), and 3(3.6%) HMKP isolates were resistant to ampicillin/sulbatam, ceftriaxone, tobramycin, and amikacin, respectively. In the present study, three HMKP isolates were resistant to all antimicrobial agents except trimethoprim-sulfamethoxazole and four carbapenem-resistant HMKP isolates were also found. The resistance rates of HMKP isolates to amicillin/sulbatam, aztreonam, ceftriaxone, ceftazidime, ciprofloxacin, levofloxacin, tobramycin, gentamicin, and trimethoprim-sulfamethoxazole were significantly lower than non-HMKP isolates (*p* < 0.05; Table 3).

**Table 3:** **The antimicrobial resistance profiling of 84 HMKP and 95 non-HMKP isolates**.

**Antimicrobials**	**HMKP (*n* = 84)**		**Non-HMKP (*n* = 95)**		***P*****-values**
	**No**.	**%**	**No**.	**%**	
Amikacin	3	3.6	7	7.4	0.254
Ampicillin	84	100.0	95	100.0	
**Amicillin/Sulbatam**	8	9.5	37	38.9	**0.000**
**Aztreonam**	6	7.1	19	20.0	**0.011**
Cefotetan	4	4.8	7	7.4	0.446
**Ceftriaxone**	7	8.3	27	28.4	**0.000**
Cefepime	4	4.8	8	8.4	0.309
**Ceftazidime**	6	7.1	17	17.9	**0.028**
**Ciprofloxacin**	5	6.0	21	22.1	**0.002**
Ertapenem	4	4.8	3	3.2	0.602
Imipenem	4	4.8	5	5.3	0.851
**Levofloxacin**	4	4.8	21	22.1	**0.002**
Nitrofurantoin	34	40.5	47	49.5	0.180
TZP^a^	4	4.8	8	8.4	0.309
Tobramycin	3	3.6	12	12.6	**0.026**
Gentamicin	5	5.9	23	24.2	**0.001**
SXT^b^	4	4.8	33	34.7	**0.000**

a *TZP: Piperacillin/tazobactam*.

b *SXT: trimethoprim/sulfamethoxazole*.

*Bold values indicate parameters with P* < 0.05 *which was considered statistically significant*.

### Prevalence of virulence-associated genes among HMKP and non-HMKP isolates

The positive rates of 17 virulence-associated genes tested among HMKP, non-HMKP, K1 and K2 isolates were showed in Table 4. The virulence-associated genes with more than 50% of positive rates among HMKP and non-HMKP isolates tested included *entB* (91.7 and 94.7%), *fimH* (77.4 and 89.5%), *uge* (90.5 and 91.6%), *ureA* (97.6 and 94.7%), *wabG* (96.3 and 94.7%), and *ycf* (90.5 and 93.7%). The positive rates of *iutA, mrkD, aerobactin, iroN*, and *rmpA* among HMKP isolates were significantly higher than those among non-HMKP isolates (*p* < 0.05). Ninety-file percentage of K1 HMKP isolates harbored *magA*, while this important virulence-associated gene was not found in any of K2 isolates, indicating that there was a strongly correlation between *magA* gene and K1 HMKP isolates (*p* < 0.01). Interestingly, *mrkD* was exclusively detected among HMKP (32.1%, 27/84) and K2 isolates (65.9%, 27/41). The positive rates of *ybtS, alls*, and *wcaG* among K1 isolates were significantly higher than those among K2 isolates (*p* < 0.01). All K5, K20, and K57 isolates were negative for *alls*. All K5 and K57 isolates were negative for *wcaG*. The positive rates of the remaining genes tested were similar between K1 and K2 isolates.

**Table 4:** **The proportions of virulence-associated genes among 84 HMKP, 95 non-HMKP, K1 and K2 isolates**.

**Genes**	**HMKP *n* = 84 (%)**	**non-HMKP *n* = 95 (%)**	***P*****-values**	**Capsular serotyping**		
				**K1 *n* = 42 (%)**	**K2 *n* = 41 (%)**	***p-*****values**
*ybtS*	43 (51.2)	45 (47.4)	0.070	**36 (78.6)**	**19 (46.3)**	**0.000**
*ureA*	82 (97.6)	90 (94.7)	0.336	41 (97.6)	40 (97.6)	0.988
*uge*	76 (90.5)	87 (91.6)	0.786	41 (97.6)	40 (97.6)	0.988
*wabG*	81 (96.4)	90 (94.7)	0.608	41 (97.6)	39 (95.1)	0.630
*ycf*	76 (90.5)	89 (93.7)	0.792	39 (92.9)	40 (97.6)	0.588
*entB*	77 (91.7)	90 (94.7)	0.572	40 (95.2)	39 (95.1)	0.975
***iutA***	**73 (86.9)**	**36 (37.9)**	**0.000**	41 (97.6)	35 (85.4)	0.124
*vatD*	5 (6.0)	1 (1.1)	0.064	1 (2.4)	1 (2.4)	1.000
*magA*	20 (23.8)	24 (25.3)	0.893	41 (97.6)	0 (0.0)	0.000
***fimH***	**65 (77.4)**	**85 (89.5)**	**0.038**	38 (90.5)	33 (80.5)	0.408
***mrkD***	**27 (32.1)**	**0 (0)**	**0.000**	**0 (0.0)**	**27 (65.9)**	**0.000**
***aerobactin***	**76 (90.5)**	**39 (41.1)**	0.000	41 (97.6)	38 (92.7)	0.558
***iroN***	**36 (42.9)**	**6 (6.3)**	**0.000**	9 (21.4)	16 (39.0)	0.053
*kfuB*	25 (29.8)	40 (42.1)	0.110	40 (95.2)	4 (97.6)	0.456
***rmpA***	**78 (92.9)**	**39 (41.1)**	**0.000**	42 (100.0)	39 (95.1)	0.342
*wcaG*	15 (17.86)	29 (30.5)	0.060	31 (73.8)	4 (9.8)	0.000
*alls*	27 (32.1)	33 (34.7)	0.930	42 (100.0)	4 (9.8)	0.000

*Bold values indicate parameters with P* < 0.05 *which was considered statistically significant*.

### Molecular characteristics of HMKP isolates

**Figure 1:**
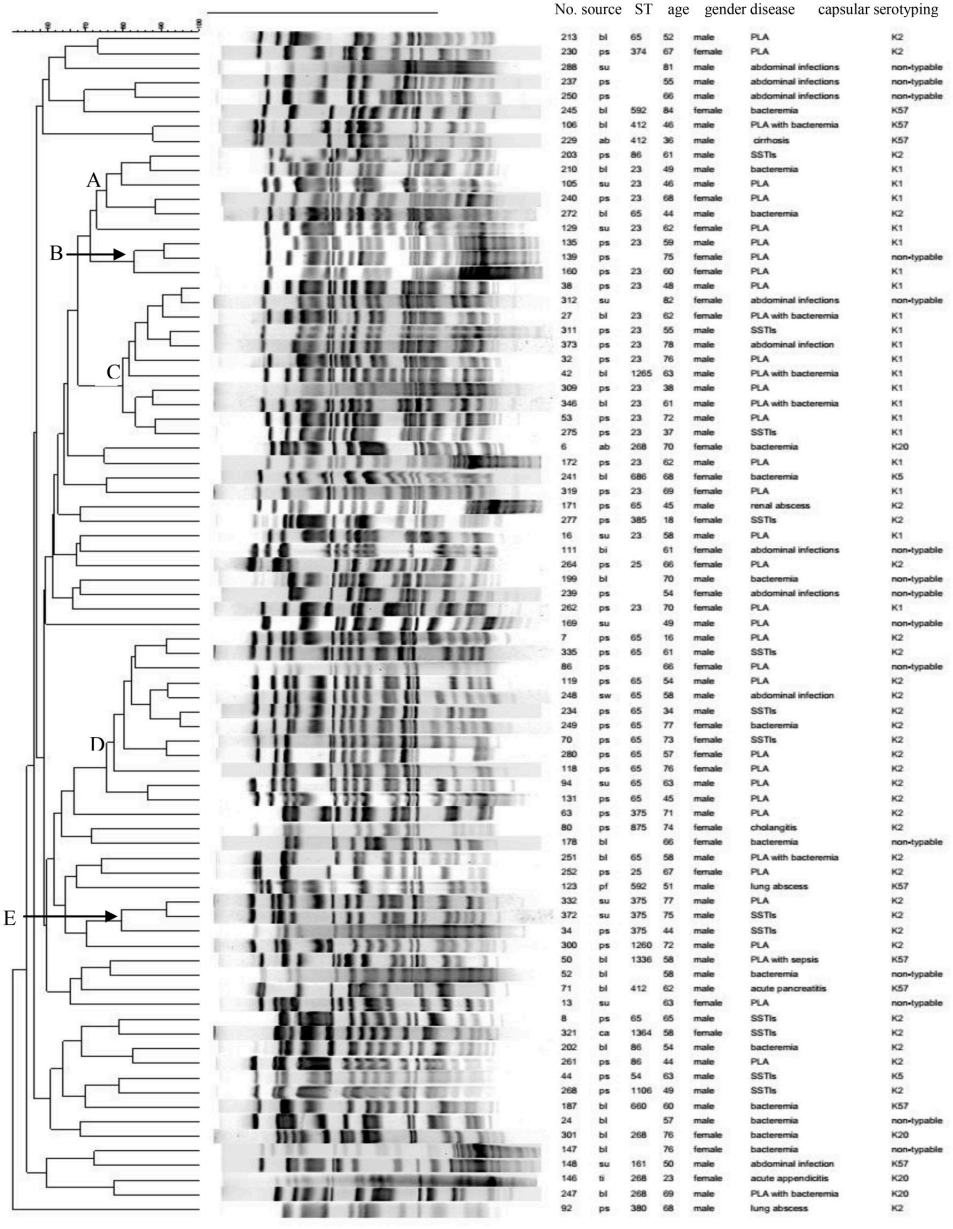
**Clonal relatedness of 84 HMKP isolates**. ps, pus; bl, blood; su, drainage; pf, pleural effusion; bi, bile; ti, tissue; ab, ascites; ca, catheter tip.

MLST was performed on the HMKP isolates with K1, K2, K5, K20, and K57 (70 isolates) and not on the K-non-typable isolates (14 isolates). Among 70 isolates tested, 21 STs were identified and one K2 isolate with seven loci not match STs in MLST database was defined as non-typable. ST23 was the most prevalent ST (27.1%, 19/70), followed by ST65 (25.7%, 18/70). The STs accounting for four isolates (5.7%, 4/70) were ST375, ST86, and ST268. ST412 was found in three isolates (4.3%, 3/70). ST25 and ST592 were identified among two isolates (2.9%, 2/70). The remaining STs were found only one isolate (Figure 1). ST23 was only found among K1 isolates, with ST23 accounting for all K1 isolates except one isolate belonging to single locus variant of ST23, ST 1265 (Figure 1). Similarly, ST65 was found exclusively among K2 isolates tested (Figure 1).

Our study also found that ST23-K1 HMKP isolates (78.9%, 15/19) were strongly associated with PLA (*p* < 0.05), while ST65-K2 isolates were correlated with more types of infections relative to ST23-K1 isolates, with 44.4% (8/18) associated with PLA. ST86, ST375, ST25, and ST380 were also exclusively identified among K2 isolates. Not only was ST268 exclusively found among K20 isolates, but also all four K20 isolates belonged to ST268. Three ST412 and two ST592 isolates belonged to K57. Among 20 K1 isolates, only ST23 (19 isolates) and its single locus variant, ST 1265 (one isolate) were identified. On the contrast, 11 STs and an unknown ST were identified among 36 K2 HMKP isolates, including ST65 (50.0%, 18/36), ST86 (11.1%, 4/36), ST375 (11.1%, 4/36), and ST25 (5.6%, 2/36; Figure 1). There were five STs including ST412, ST592, ST1336, ST161, and ST660 among eight K57 isolates. Two STs, ST54, and ST686, were found among two K5 isolates. Nine isolates causing SSTIs belonged to six STs (ST54, ST23, ST375, ST65, ST86, and ST1106). Six STs (ST268, ST412, ST161, ST23, ST875, and ST65) were found among eight MLST-typed isolates which were associated with abdominal infections. Among 16 isolates typed by MLST which were associated with bacteremia, 10 STs were identified, with no prevalent ST. Although 12 STs were found among 34 MLST-typed isolates causing PLA, 44.1% (15/34) and 23.5% (8/34) belonged to ST23-K1 and ST65-K2.

PFGE results showed that the homology of 84 HMKP isolates was diverse (Figure 1). Only five PFGE clusters with more than 75% similarity accounted for more than three isolates. These five PFGE clusters only accounted for 35 (41.7%, 35/84) isolates. PFGE cluster A accounted for six isolates including four ST23-K1, one ST65-K2, and one ST86-K2 isolate. PFGE cluster B accounted for three isolates including two ST23-K1 and one K-non-typable isolate. PFGE cluster C accounted for 11 isolates including nine ST23-K1, one ST1265-K1, and one K-non-typable isolate. PFGE cluster D accounted for 12 isolates including 11 ST65-K2 and one K-non-typable isolate. PFGE cluster E accounted for three ST375-K2 isolates.

## Discussion

A recent multicenter study from China showed that the prevalence of HMKP among *K. pneumoniae* isolates causing various types infections including PLA, bloodstream infections, hospital-acquired pneumonia, and intra-abdominal infections in10 cities of China during February to July 2013 were 37.8% (Zhang et al., 2016). Another Chinese study conducted in a single center in Beijing, China, showed that the prevalence of HMKP was 33% (Yang et al., 2014). Yan et al. reported that 14 (28.6%) of 49 *K. pneumoniae* isolates associated with ventilator-associated pneumonia from a university hospital in China from January 2014 to December 2014 were HMKP (Yan et al., 2016). The HMKP prevalence of our study was lower than those Chinese reports mentioned above, but significantly higher than the reports from a teaching hospital in Spain between 2007 and 2013 (5.4%, 53/878; Cubero et al., 2016) and a surveillance study in Alberta, Canada (8.2%; Peirano et al., 2013). Sun et al. reported 81.6% (31/38) of *K. pneumoniae* causing PLA were HMKP determined by the string test (Sun et al., 2016), which was significantly higher than that in our investigation. A report from Taiwan also showed that 90% of *K. pneumoinae* associated PLA were HMKP (Yu et al., 2008). However, in another Chinese study, only 28.9% (13/45) of *K. pneumoniae* isolates causing PLA were HMKP exhibiting hypermucoviscosity phenotype (Qu et al., 2015), which was lower than that in the present study. These data indicated that the proportion of PLA caused by HMKP showed a varied geographic distribution. Li et al. found that neither age nor sex was associated with hypermucoviscosity phenotype determined by string test (Yang et al., 2014). Zhang et al. also found age and sex were not correlated with HMKP (Zhang et al., 2016). However, HMKP prevalence in male ≤60-year old patients was significantly higher than that in female ≤60-year old patients in the present study. Our further investigation indicated that male patients with 41–50 years predisposed to HMKP infections. Diabetes mellitus has been considered as a significant risk factor for HMKP infection (Cheng et al., 1991; Wang et al., 1998; Shon et al., 2013; Zhang et al., 2016). However, other studies did not found this correlation (Yu et al., 2007; Liu et al., 2014; Yang et al., 2014; Yan et al., 2016). Zhang et al. reported that the proportion (33.8%) of patients with cancer among patients with HMKP infections was significantly higher than that (18.8%) among patients with cKP infections and showed that cancer (OR = 2.285) appeared to be independent variable associated with HMKP by multivariate analysis. However, in the present study, only two patients (2.4%) with HMKP infections were found to have solid tissue cancer while 9.2% of patients with non-HMKP infections had solid tissue cancer. Interestingly, we also found that there was an association between hypertension and HMKP infections (11.9 vs. 0.70%) and hypertension (OR = 7.333) was an independent risk factor for HMKP infections determined by multivariate regression analysis. Leukemia (OR = 0.190) was negatively correlated with HMKP infections (*p* < 0.05) determined by univariate analysis, but not by multivariate analysis.

Although HMKP isolates are usually more susceptible to clinically often used antimicrobial agents relative to non-HMKP isolates, more and more HMKP isolates were found to be multi-resistant to antimicrobial agents, even to carbapenems (Yang et al., 2014; Yao et al., 2015; Wei et al., 2016). In the present study, four HMKP isolates were found to be resistant to carbapenem. Acquisition of hyper virulence and carbapenem resistance poses major problems in the management of *K. pneumoniae* infection.

In contrast to previous reports with K1 being the most prevalent capsular serotype among HMKP isolates (Liu et al., 2014; Yang et al., 2014; Qu et al., 2015; Yan et al., 2016; Zhang et al., 2016), our study showed that K2 was the most common capsular serotype and K2 HMKP isolates were associated with more types of invasive infections than K1 isolates. In the present study, 95.2% of *K. pneumoniae* isolates with hypermucoviscosity phenotype were positive for *rmpA*, especially all K1, K2, K5, K20, and K57 isolates positive for *rmpA*, which was similar to a multicenter investigation from China (97.7%; Zhang et al., 2016), but was significantly higher than another report from Beijing, China (55%; Zhang et al., 2016). Surprisingly, all 14 isolates with K-non-tyable capsular serotypes were negative for *rmpA*. Previous studies showed that there was a correlation between *rmpA* gene and virulence in terms of abscess formation for HMKP isolates (Yu et al., 2006; Yang et al., 2014; Yan et al., 2016; Zhang et al., 2016). However, a recent study from China showed that all *K. pneumoniae* isolates causing PLA were positive for *rmpA*, regardless of hypermucoviscos phenotype(Qu et al., 2015). In our investigation, 41.1% of non-HMKP isolates were found to be positive for *rmpA*. The hypermucoviscosity phenotype in *K. pneumoniae* isolates is associated with the carriage of chromosomally encoded *magA*, which is characteristic of the K1 capsular operon (Fang et al., 2004, 2005; Chuang et al., 2006). *magA* has been described as the causative gene for *K. pneumoniae* isolates associated with PLA and septic metastatic complications(Fang et al., 2005; Chuang et al., 2006). Yan et al. reported that all *K. pneumoniae* isolates including HMKP and non-HMKP causing ventilator-associated pneumonia were positive for *mrkD* (Yan et al., 2016). Another study showed that 96.3% of *K. pneumoniae* isolates were positive for *mrkD* (El Fertas-Aissani et al., 2013). However, our study found that *mrkD* was only found among HMKP isolates and only among K2 isolates. To the best of our knowledge, this is the first report of the association between *mrkD* and K2 HMKP isolates. Our study also showed *alls, ybtS*, and *wcaG* were correlated with K1 isolates. Although Yu et al. reported that there was a strong association between *kfuB* and *alls* and K1 isolates, with all K1 isolates positive for *kfuB* and *alls* and all K2 isolates negative for these two genes (Yu et al., 2008), *kfuB* and *alls* were found among 8.5 and 11.1% of K2 HMKP isolates in the present study. *aerobactin* is important virulence determinant for hvKP even used as the marker for the identification of hvKP, instead of hypermucoviscosity phenotype determined by the string test (Zhang et al., 2016). In the present study, 95.1 and 41.1% of HMKP and non-HMKP isolates were found to harbor *aerobactin*, indicating that hypermucoviscosity phenotype as the marker for the identification of hvKP is controversial.

ST23 has been found to be the most commonly described ST among HMKP isolates and is strongly correlated with capsular serotype K1 and liver abscess (Turton et al., 2007; Chung et al., 2008; Shon et al., 2013). Similar to previous reports, our data showed that ST23 was the most prevalent among K1 isolates. ST57 and ST82 are also found to be associated with the K1 serotype and PLA (Brisse et al., 2009; Merlet et al., 2012). However, these two STs were not found in the present study. Previous studies showed that ST268 was only found among K20 isolates (Liu et al., 2014; Lin et al., 2015; Yan et al., 2015, 2016; Zhang et al., 2016). In the present study, we also found that there was an association between ST268 and K20 isolates. Eight different STs including ST65, ST66, ST86, ST373, ST374, ST375, ST380, and ST434 was found among capsular serotype K2 isolates from Singapore, Hong Kong, and Taiwan (Lin et al., 2014). Eleven STs were identified among K2 isolates associated with different types of infections from Taiwan, among which ST65 was the most common (*n* = 10), followed by ST86, ST373, and ST375 (Liao et al., 2014). In the present study, 16 (80%) of 20 K1 isolates belonged to PFGE cluster B or C and no K1 isolates belonged to PFGE cluster A, D, E, F, or G. However, K2 isolates belonged to more PFGE clusters including cluster A, B, D, E, and F. Our data support further the evidence that the composition of ST types and PFGE clusters among *K. pneumoniae* K2 isolates was more diverse than K1 isolates. Our data also indicated that the distributions of STs among HMKP isolates causing bacteremia, abdominal infections and SSTIs were more diverse than that associated with PLA.

In conclusion, our study first found that hypertension and male patients with 41–50 years old were independent risk factors. The composition of ST types and PFGE clusters among *K. pneumoniae* K2 isolates was more diverse than K1 isolates. K1 and K2 HMKP isolates had respective specific profiles of virulence-associated genes.

## Author contributions

YG, SW, LZ, YJ, JD, ZH, JL, XQ isolated bacteria and performed the laboratory measurements. FY and LW made substantial contributions to conception and design. LC and BK revised the manuscript critically for important intellectual content. LC and JL participated in experimental design and data analysis. FY drafted the manuscript. All authors read and approved the final manuscript.

### Conflict of interest statement

The authors declare that the research was conducted in the absence of any commercial or financial relationships that could be construed as a potential conflict of interest.
